# Rational Redesign of Glucose Oxidase for Improved Catalytic Function and Stability

**DOI:** 10.1371/journal.pone.0037924

**Published:** 2012-06-13

**Authors:** J. Todd Holland, Jason C. Harper, Patricia L. Dolan, Monica M. Manginell, Dulce C. Arango, Julia A. Rawlings, Christopher A. Apblett, Susan M. Brozik

**Affiliations:** Biosensors & Nanomaterials, Sandia National Laboratories, Albuquerque, New Mexico, United States of America; Centro de Biología Molecular Severo Ochoa (CSIC-UAM), Spain

## Abstract

Glucose oxidase (GOx) is an enzymatic workhorse used in the food and wine industries to combat microbial contamination, to produce wines with lowered alcohol content, as the recognition element in amperometric glucose sensors, and as an anodic catalyst in biofuel cells. It is naturally produced by several species of fungi, and genetic variants are known to differ considerably in both stability and activity. Two of the more widely studied glucose oxidases come from the species *Aspergillus niger* (*A. niger*) and *Penicillium amagasakiense* (*P. amag.*), which have both had their respective genes isolated and sequenced. GOx from *A. niger* is known to be more stable than GOx from *P. amag.*, while GOx from *P. amag*. has a six-fold superior substrate affinity (*K*_M_) and nearly four-fold greater catalytic rate (*k*_cat_). Here we sought to combine genetic elements from these two varieties to produce an enzyme displaying both superior catalytic capacity and stability. A comparison of the genes from the two organisms revealed 17 residues that differ between their active sites and cofactor binding regions. Fifteen of these residues in a parental *A. niger* GOx were altered to either mirror the corresponding residues in *P. amag*. GOx, or mutated into all possible amino acids via saturation mutagenesis. Ultimately, four mutants were identified with significantly improved catalytic activity. A single point mutation from threonine to serine at amino acid 132 (mutant T132S, numbering includes leader peptide) led to a three-fold improvement in *k*_cat_ at the expense of a 3% loss of substrate affinity (increase in apparent *K*_M_ for glucose) resulting in a specify constant (*k*_cat_/*K*_M_) of 23.8 (mM^−1^ · s^−1^) compared to 8.39 for the parental (*A. niger*) GOx and 170 for the *P. amag.* GOx. Three other mutant enzymes were also identified that had improvements in overall catalysis: V42Y, and the double mutants T132S/T56V and T132S/V42Y, with specificity constants of 31.5, 32.2, and 31.8 mM^−1^ · s^−1^, respectively. The thermal stability of these mutants was also measured and showed moderate improvement over the parental strain.

## Introduction

We selected GOx (1.1.3.4) for rational redesign employing genetic engineering techniques for improvement of specific activity and affinity towards the substrate without sacrificing stability. GOx, produced primarily by filamentous fungi such as *Aspergillus niger* (*A. niger*) and *Penicillium amagasakiense* (*P. amag.*), is a holoenzyme consisting of two identical 80 kDa subunits which require a cofactor (flavin adenine dinucleotide, FAD) for activity. Although much work has been done in the field of directed enzyme evolution in general [1], [2], [3], [4], [5], [6], [7], [8], there are few reports on the genetic manipulation of GOx [9], [10], [11]. Zhu et al. employed directed evolution to generally enhance the catalytic performance of GOx resulting in a 1.5 fold improvement in *k*_cat_ [9]. In a subsequent work, Zhu and colleagues again utilized directed evolution to improve the performance of GOx at electrical anodes in conjunction with the mediator ferrocene-methanol [10]. Chen and coworkers genetically engineered GOx to include a poly-lysine ‘tether’ in order to anchor more ferrocenecarboxylic acid mediator to the enzyme resulting in improved stability and sensitivity in a glucose biosensor utilizing the engineered GOx [11].

In this work a comparison of the sequence and structure-function similarity in GOx and enzymes homologous to it from various organisms was conducted in order to identify sequence variations within conserved regions across organisms which could provide logical targets for mutagenesis. This design stage was performed using the BLASTP search engine to search NCBI’s non-redundant protein database [12] for homologous proteins, and sequence alignments were made using CLUSTALW. Through sequence analysis, it was found that there is a high degree of conservation in the FAD- and substrate-binding domains among glucose oxidases. Among the two most studied GOx enzymes, it was previously noted that while *A. niger* GOx is more stable than GOx from *P. amag.,* the latter enzyme has a six-fold higher substrate affinity (reflected as a lower *K*_M_) and nearly four-fold higher catalytic rate (*k*_cat_) at pH 7 [13], [14].

**Figure 1:**
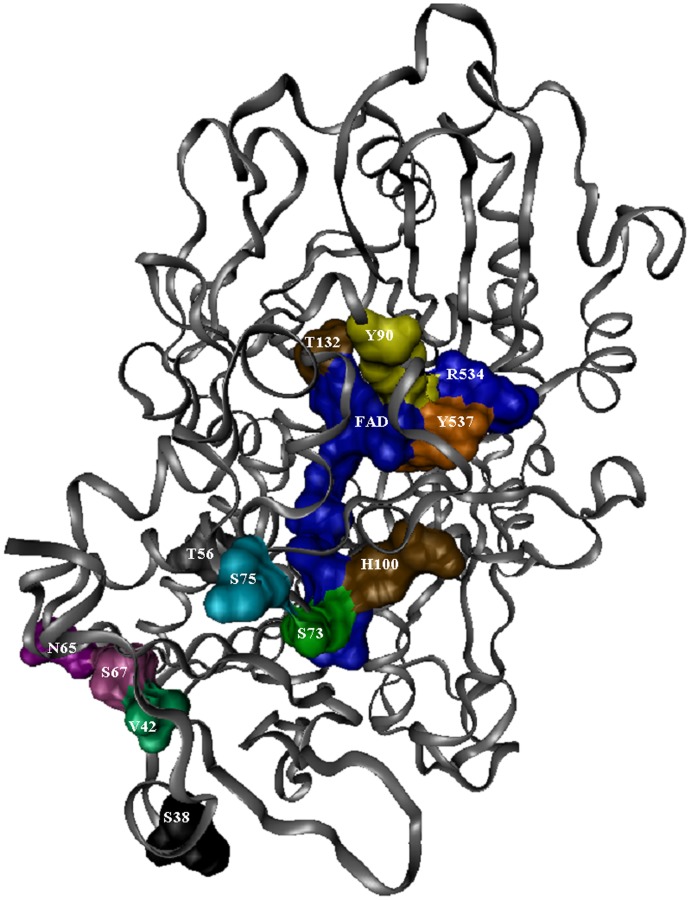
A cartoon view of GOx monomer, with the protein shown as ribbons and the FAD groups shown as space-filling models. Residues that were targeted for mutagenesis are also shown as space-filling models and labeled. The numbering used is from the *A. niger* protein sequence and includes the 22 amino acid leader peptide. Residue N536 is obscured by residues T132, R534, and T537.

Based on these observations, 15 amino acids in the FAD- and substrate-binding sites of *A.* niger were selected for site-directed mutagenesis to mirror the comparable residues from *Penicillium*, or for saturation mutagenesis. In the first region of the FAD-binding domain, the site of ADP-binding and the most conserved region of the FAD-binding site, nine residues are not conserved between *A. niger* and *P. amag*. Eight were targeted for site-directed mutagenesis as follows (*A. niger* sequence, numbering used in this paper includes the leader peptide): serine to alanine in position 38 (S38A), V42T, T56V, N65K, S67K, S73K, S75F, and H100Q. The active site of GOx is formed by tyrosines (Y90 and Y537), threonine (T132), arginine (R534), asparagine (N536), and two histidine residues (H538 and H581) (Meyer et al., 1998). With the exception of the two histidines, none of these residues is conserved across the glucose-methanol-choline (GMC) oxidoreductase family [15]. This region is therefore the most heterogeneous region among the GMC oxidoreductases, and was targeted for saturation mutagenesis, with the exception of tyrosine 537, which was only changed to tryptophan to mirror the *P. amag.* structure. All of the mutations, both site-directed and saturation, were initially done independently of each other. **Figure 1** illustrates the position of these residues in relation to the cofactor and substrate binding sites. Once these mutants were created, a colorimetric kinetic assay was used to determine the viability of the variant enzymes. Promising mutants were then characterized more extensively using an electrochemical assay. The most promising mutants were then subjected to further rounds of saturation mutagenesis and combined with each other into double mutants via site-directed mutagenesis.

**Figure 2:**
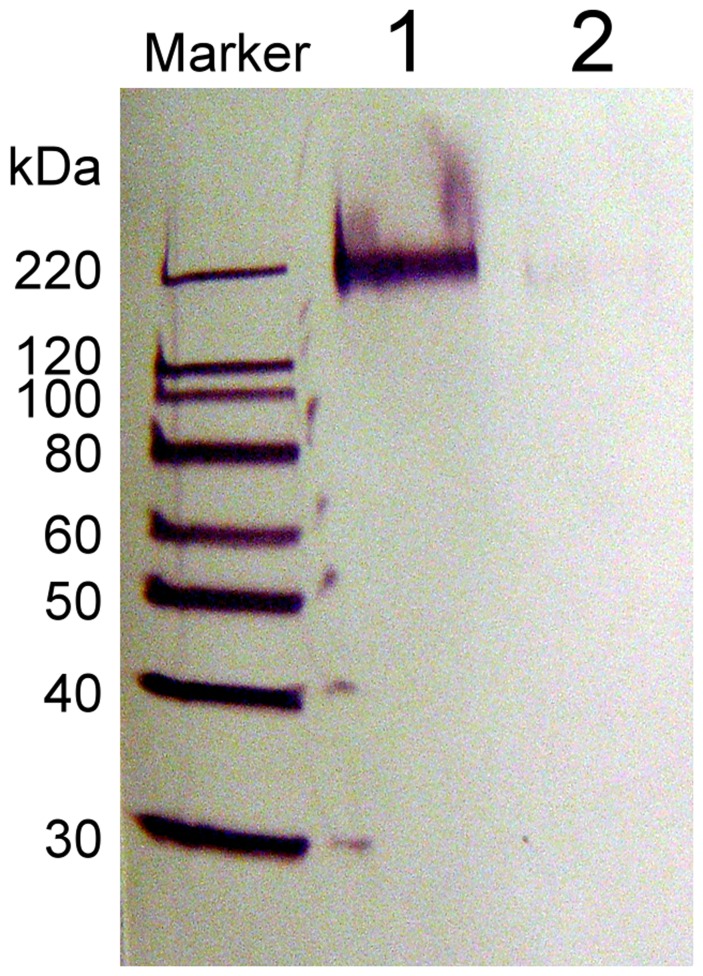
Western blot of Ni-NTA affinity purified yeast culture media. Bands observed via anti-V5 epitope-AP antibody labeling. Lane 1: Sample from culture with induced GOx expression; Lane 2: Sample from uninduced culture.

## Materials and Methods

### Site-directed and Saturation Mutagenesis

A synthetic version of the *A. niger* GOx gene, designed for optimized codon efficiency in *Saccharomyces cerevisiae (S. cerevisiae)*, was constructed by DNA 2.0 (Menlo Park, CA). The pYES-DEST52 Gateway® Vector (Invitrogen, Carlsbad, CA) was used for cloning the GOx gene into yeast. This galactose-inducible *S. cerevisiae* expression vector includes a c-terminal hexa-histidine tag and a V5 antibody epitope. The native amino-terminal signal peptide sequence flagging GOx for secretion was preserved. The recombinant vector was then used to transform the *S. cerevisiae* strain INV*Sc*1 (*MATa his3D1 leu2 trp 1-289 ura3-52*; Invitrogen). Mutations to this construct were made using the QuikChange® Multi Site-Directed Mutagenesis Kit (Stratagene, La Jolla, CA). Saturation mutagenesis was achieved by using mixtures of mutagenic oligonucleotides (degenerate oligos).

**Figure 3:**
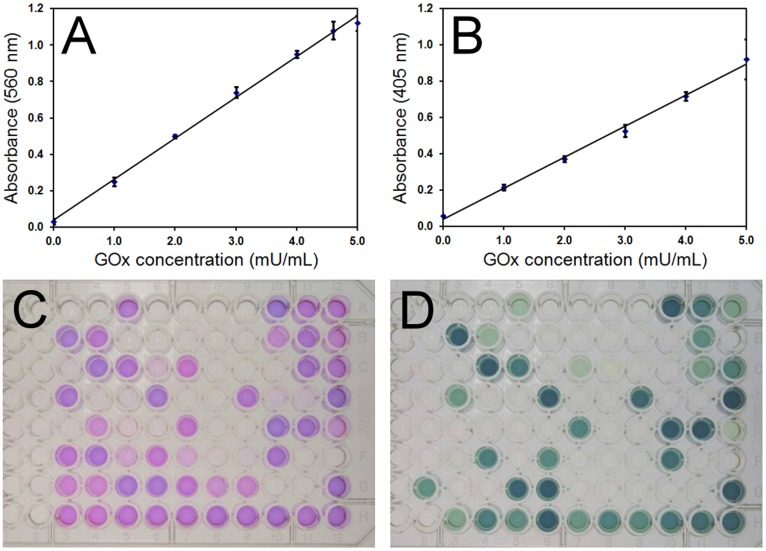
Comparison between Amplex Red and ABTS GOx activity assays. A) Amplex Red and B) ABTS assay absorbance vs. GOx concentration standard curves. Glucose concentration was 50 mM. Error bars are the standard deviation of 3 independent measurements. Comparison of activity assay results for mutant GOx stains using C) Amplex Red or D) ABTS assay. Each 96 well plate contained 96 different mutant GOx samples that were loaded in identical wells between plates.

### Protein Isolation and Purification

Purification of the secreted recombinant GOx was achieved by using a tetradentate nickel chelate (Ni-NTA) purification system (Invitrogen). After 24-hour growth in 50 mL of YP-Gal induction media, the soluble His-tagged GOx was harvested by centrifugation at 1400×g. The media was subsequently dialyzed with 50 mM NaH_2_PO_4_ buffer, pH 8.0, to remove residual amino acids. Purification was carried out under native conditions as described in the manufacture’s protocol with slight modifications. Briefly, 1 mL of Ni-NTA resin was added to polypropylene columns, washed and then equilibrated with 50 mM NaH_2_PO_4_ buffer containing 20 mM imidazole (native binding buffer). Concentrated GOx samples were diluted to 6 mL volumes using 3 mL 18 MΩ water (Barnstead NanoPure water purifier, Boston, MA) followed by 3 mL of native binding buffer. Samples were incubated for 1 hour at 25°C with gentle mixing. After washes, the purified GOx was eluted off the resin using 1.5 mL of an elution buffer consisting of 50 mM NaH_2_PO_4_, 250 mM imidazole, pH 5.0. The resin was incubated in the elution buffer for 15 minutes with mixing.

**Figure 4:**
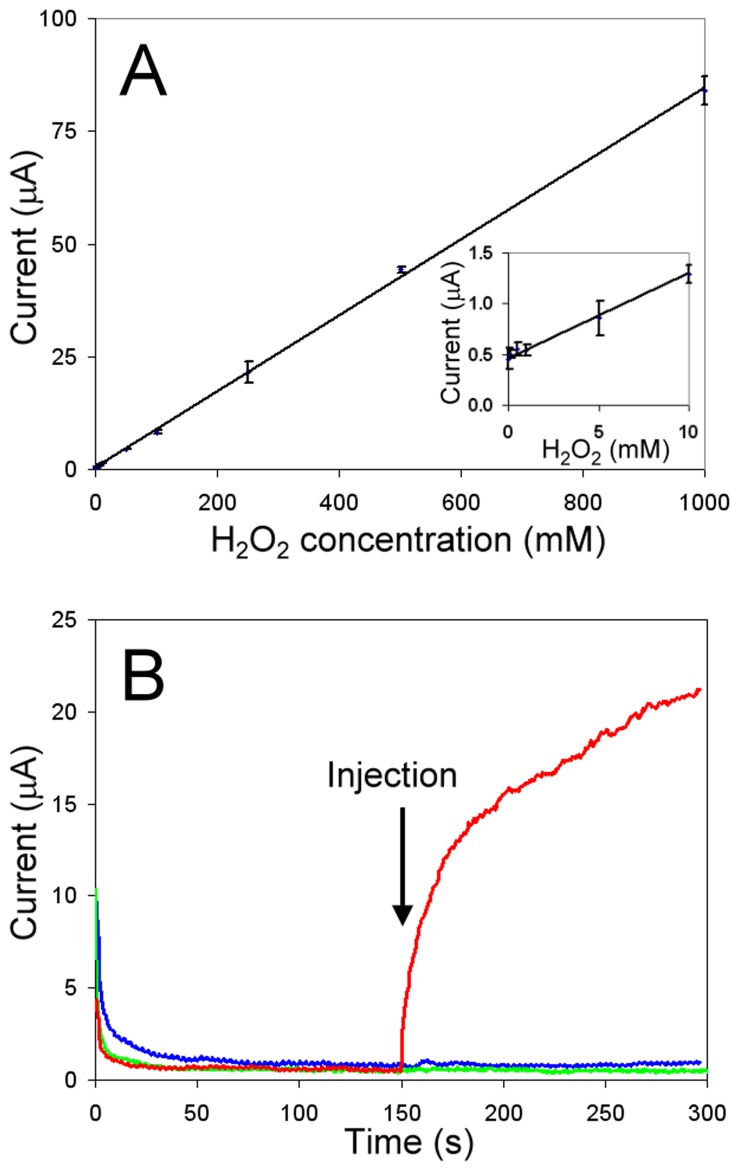
Electrochemical GOx activity assay: H_2_O_2_ concentration calibration and assay controls. A) Steady state current response vs. H_2_O_2_ concentration. Pt working electrode held at +700 mV vs. Ag/AgCl, 6000 rpm, in 100 mM NaPB, pH 6.8. Error bars are the standard deviation of 3 independent measurements. Inset: Lower H_2_O_2_ concentration segment of plot. B) Current response upon a 25 µL injection of sample prepared from yeast cultures in which GOx expression was induced (red trace) or uninduced (blue trace), into 100 mM NaPB, pH 6.8 with 100 mM glucose. Injection of sample prepared from induced GOx expression yeast culture into 100 mM NaPB, pH 6.8, without glucose (green trace). Pt working electrode held at +700 mV vs. Ag/AgCl, 6000 rpm.

The presence of the hexa-histidine and V5 epitope tags allowed for the use of affinity chromatography and immunodetection as tools to evaluate the presence of recombinant GOx in the purified sample. Post-purification concentration was measured and an aliquot was removed for SDS-polyacrylamide gel electrophoresis (SDS-PAGE). Samples were run on 10–20% Tris-Glycine gels for 1 hour. Proteins were then transferred onto a nitrocellulose membrane followed by immunodetection using an anti-V5 (C-term)-AP antibody and a chromogenic immuno-detection kit (WesternBreeze, Invitrogen). After blocking with a buffer saline-detergent-casein solution (supplied) the membrane was incubated with 15 mL of a 1∶2000 dilution of anti-V5 (C-term)-AP primary antibody. An alkaline phosphatase (AP) conjugated anti-IgG antibody (supplied) was used to visualize the Magic Mark Protein standard ladder. After washing, the membrane was incubated in 10 ml of BCIP/NBT chromogenic substrate for alkaline phosphatase.

**Table 1:** Kinetic rate parameters of parental and mutant GOx stains for D (+) glucose oxidation as determined via initial rate electrochemical measurements.

				Improvement over Parent	
GOx Strain	*K*_M_ (mM)^1^	*k*_cat_ (s^−1^)^1,2^	*k*_cat_/*K*_M_ (s^−1^ ⋅ mM^−1^)	(% *K*_M_)^3^	(×*k*_cat_)
				–	–
*P. amag.* 4	6.4	1085	170		
T132S	34.5±6.8	821±52	23.8	+3.6	2.9
T132S/T56V	20.8±6.0	670±54	32.2	−38	2.4
T132S/V42Y	26.3±6.9	838±67	31.8	−21	3.0
V42Y	40.2±8.3	1264±87	31.5	+21	4.5

1 ± values are 95% confidence intervals from a non-linear least squares regression fit of initial rate data to the Michaelis-Menton equation.

2 *k*_cat_ defined per mol native GOx.

3 Negative change in *K*_M_ denotes higher affinity for substrate.

4 Values taken from reference 14.

### Sample Preparation for Activity Assays

Each clone was inoculated into 50 mL of YP-Gal induction media and incubated for 24 hours at 30°C. To prepare recombinant GOx secreted into spent growth media for activity assay, Then the cultures were briefly centrifuged to pellet the cells, which were then discarded. The collected media was then filtered and concentrated using 30 kDa molecular weight cut-off (MWCO) Centricon Plus-70 centrifugal filters (70 mL processing vol., Millipore, Billerica, MA) to an end volume of 0.5 mL. The retained high mass fraction was then buffer exchanged with 50 mM NaH_2_PO_4_ again using 30 kDa MWCO centrifugal filers. GOx concentration in filtered, concentrated, and buffer exchanged culture media samples was determined via SDS-PAGE against known concentrations of GOx from *A. niger.* using Sypro Ruby Red (Molecular Probes, Eugene, OR) as protein stain. An immunoaffinity assay was used to determine the band that corresponded to GOx (as above), and ImageQuant software (Amersham Biosciences, Piscataway, NJ) was used to correlate band intensity to protein concentration.

**Table 2:** Thermal stability of parental and mutant GOx stains incubated at 50 °C.

GOx Strain	*T*_70%_ (hrs)^1^	*T*_1/2_ (hrs)^1^	Corr. Coeff. (R^2^)^2^
Biozyme	10.4±0.5	20.1±0.9	0.990
Parent	14.8±1.1	28.8±1.9	0.992
T132S	19.8±1.3	38.5±2.4	0.986
T132S/T56V	20.4±1.0	39.6±2.4	0.981
T132S/V42Y	17.4±0.7	33.8±2.5	0.974
V42Y	20.7±1.1	40.3±2.3	0.991

1 Error corresponds to the standard deviation of GOx kinetic rate measurements performed in triplicate.

2 Correlation coefficients obtained from an exponential least squares regression fit of GOx kinetic rates measured in triplicate vs. time.

### Colorimetric Enzyme Activity Assay

Amplex Red Glucose Oxidase Assay (Invitrogen) was used according to the manufacturer’s protocol. A second colorimetric assay method using 2, 2′-azino-bis [3-ethyl-benzothiazoline-6-sulfonic acid] (ABTS, Thermo Scientific, Waltham, MA) was modified from Sun, et al [16]. The ABTS working solution consisted of 100 mM sodium phosphate buffer, pH 7.0, 50 mM glucose, 3 units of horseradish peroxidase (HRP), and 2 mg/mL ABTS. The reaction solution in both cases consisted of 50 µL of protein sample combined with 50µL of working solution. Absorbance measurements were taken in 96 well plates ten seconds after mixing, and were done in triplicate.

**Figure 5:**
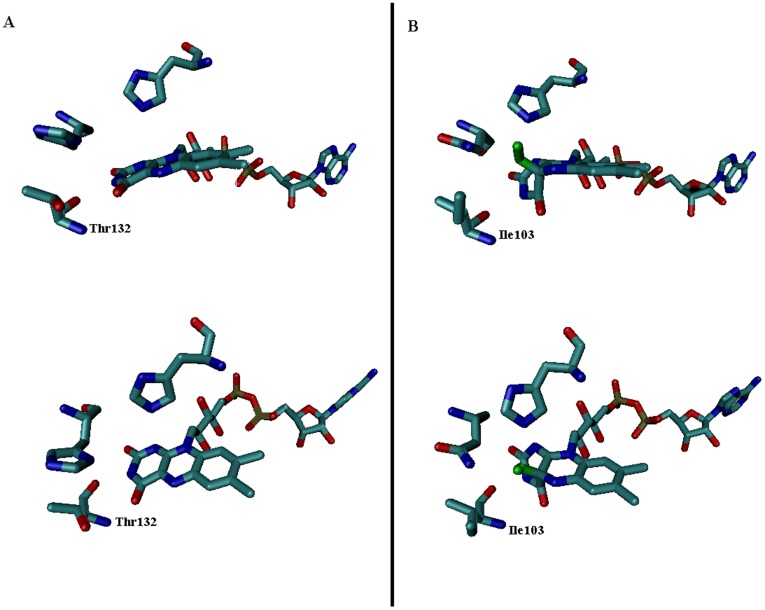
Structural comparison of the GOx FAD adduct (1cf3) with the FAD-peroxy adduct of choline oxidase (2jbv). Panel A has illustrations of GOx at two different angles. Panel B illustrates the choline oxidase FAD-peroxy adduct at two different angles. Thr 132 (GOx) and its homologous amino acid Ile 103 (choline oxidase) are labeled. The peroxy adduct is colored green. Other oxygens are red, carbons are teal, nitrogens are blue, and phosphorous atoms are tan.

**Figure 6:**
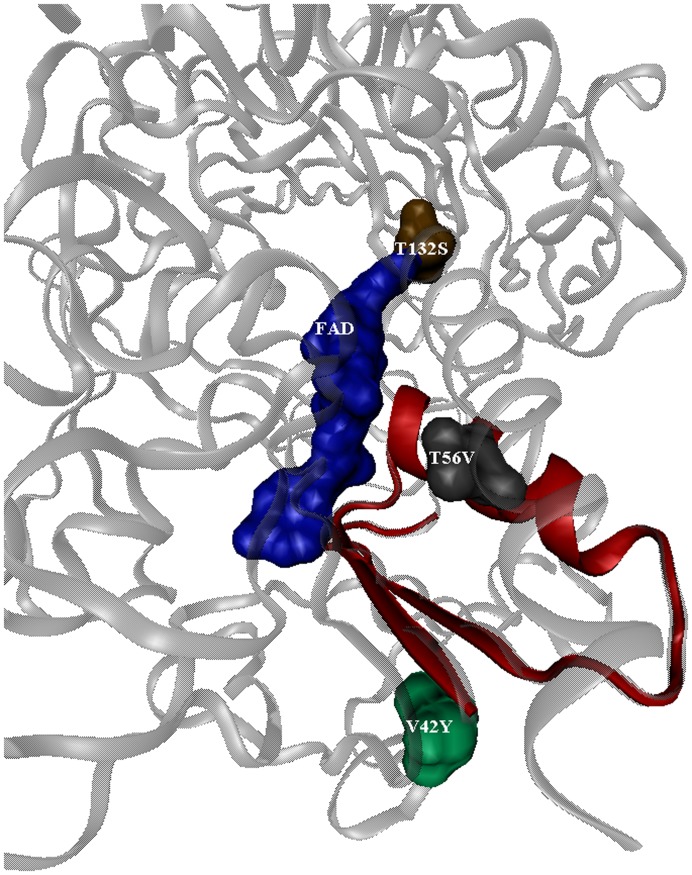
View of amino acid mutations that enhanced GOx kinetic activity in relation to the FAD cofactor: T132S, T56V, and V42T. The monomer protein is shown as ribbons. The FAD group and mutated amino acid residues are shown as space-filling models. The FAD binding peptide region, containing amino acids T56V and V42T, is colored red.

### Electrochemical Enzyme Activity Assay

Electrochemical initial rate measurements were performed on a PGZ 402 VoltaLab potentiostat (Radiometer Analytical, Lyon, France) and were measured versus a micro Ag/AgCl reference (Microelectrodes Inc, Bedford, NH) and a platinum (Pt) wire counter electrode. A rotating disk electrode system (RDE-1, Bioanalytical Systems, West Lafayette, IN) was used in conjunction with the 3 mm diameter Pt working electrode (Bioanalytical Systems). Polystyrene 24 well plates (3.5 mL well volume) were used as the electrochemical cell (BD Falcon, San Jose, CA).

Prior to each initial rate assay the Pt electrode was thoroughly cleaned to ensure a consistent electrode surface. The Pt electrode was rinsed in 18 MΩ water followed by electrochemical cleaning via potential cycling in 1 M H_2_SO_4_ from −200 to +1200 mV vs. Ag/AgCl at 100 mV s^−1^ until a stable voltammogram was obtained with well defined hydrogen and oxygen absorption and desorption waves (typically 25 cycles). Afterwards the electrode was thoroughly rinsed and sonicated for several seconds in 18 MΩ water. The electrode was further conditioned in 1 mM ferricyanide, 1 mM ferrocyanide, 0.1 M KCl, in 10 mM sodium phosphate buffer (NaPB), pH 7.0, via 0.25 second chronopotentiometric steps from −0.5 µA to +0.5 µA for a total of 500 cycles. This was followed by 10 cyclic voltammograms in the same solution from −100 mV to +500 mV at 100 mV s^−1^. The anodic and cathodic potential peak separation (?*E*_p_) of the final sweep was used to determine whether the electrode had been adequately cleaned. If the ?*E*_p_ exceeded 80–90 mV, the cleaning process was repeated starting with H_2_SO_4_ cleaning. Following final conditioning, the electrode was thoroughly rinsed in 18 MΩ water.

For enzymatic activity assay, the Pt electrode was immersed with the reference and counter electrodes in 1.775 mL of 100 mM NaPB, pH 6.8, containing a given concentration of glucose. The Pt electrode was rotated at 6000 rpm and a chronoamperometric step to +700 mV vs. Ag/AgCl was applied. The system obtained a pseudo steady state current response within 150 seconds after which 25 µL of the enzyme solution was injected into the well.

### Stability Assay

To determine the thermal stability of the mutant and commercial enzymes, tubes of enzyme were incubated in the ABTS assay buffer at 50°C for 48 hours. Every two hours an aliquot was removed from each tube and an ABTS kinetic assay was performed, as described above. Absorbance measurements were taken every minute for ten minutes, and each set of measurements was done in triplicate.

## Results

### Protein Isolation and Purification

Recombinant GOx secreted by yeast into culture media was harvested, affinity purified, ran on a SDS-PAGE gel, and visualized by Western blotting with anti-V5-AP antibodies using the WesternBreeze Chromogenic Immuno-detection, as shown in **Figure 2**. Hyperglycosylated recombinant GOx was positively identified at ∼180 kDa. Colorimetric ABTS assays performed directly in native gels also confirmed the identity of this band as GOx (data not shown). Expression in *E. coli* was abandoned due to the formation of inclusion bodies, and the difficulty in reconstituting the functional enzyme from them.

### Colorimetric Activity Assays

GOx catalyzes the oxidation of β-D-glucose to D-glucono-1,5-lactone and hydrogen peroxide, using molecular oxygen as the electron acceptor as shown in the following two half-reactions:

*β*-D-glucose + GOx:FAD + H_2_O ↔ GOx:FADH_2_+ gluconolactone +2H^+^(1)

GOx:FADH_2_+ O_2_+2H^+^ → GOx:FAD + H_2_O_2_(2)

In the interest achieving higher throughput, in all assays concentrated, buffer exchanged, partially purified protein from spent culture media was used instead of protein purified to homogeneity via nickel-affinity column chromatography. Using immunoaffinity labeling, band in SDS-PAGE gels corresponding to GOx in such samples was clearly identified. Protein concentration was quantified from the band intensity using ImageQuant software (Amersham Biosciences, Piscataway, NJ) as described in the Methods section. All measurements were performed in triplicate to insure accuracy and reproducibility.

Two different assay methods were used for screening of mutants: colorimetric and electrochemical. Both methods rely on the generation of hydrogen peroxide produced as a by-product of GOx turnover. Though the optimal pH for both the *A. niger* and *P. amag.* enzymes is near 6, both retain nearly 90% activity at pH 7 [13], [14]. Since many applications for GOx are conducted near physiological pH values, we carried out all of our assays at either pH 6.8 (electrochemical) or 7.0 (colorimetric) to insure that our enzymes would be useful under such conditions.

The Amplex Red Glucose Oxidase Assay provides a sensitive coupled enzyme method for the detection of GOx activity based on peroxidase turnover of hydrogen peroxide produced by GOx in the presence of the Amplex Red reagent (10-acetyl-3,7-dihydroxyphenoxazine). For the Amplex Red GOx assay, a standard curve with GOx concentrations ranging from 0–5 mU/ml was constructed and is presented in **Figure 3A**. A linear dependence of absorbance on GOx concentration was observed over the conditions tested with high reproducibility between measurements (R^2^ = 0.9971). The ABTS colorimetric assay also relies on the coupled GOx-peroxidase turnover yielding polymerized ABTS and a standard curve with GOx concentrations ranging from 0–5 mU/ml was also generated using this assay (**Figure 3B**). Similar to the Amplex Red assay, the ABTS assay yielded a strong linear dependence of absorbance on GOx concentration with high reproducibility (R^2^ = 0.9964). The Amplex Red assay exhibited a higher sensitivity of 0.224 (Abs a.u./mU GOx) compared with 0.171 measured from the ABTS assay.

When employing the Amplex Red and ABTS GOx activity assays to evaluate the activity of GOx mutants, disagreement between the two different colorimetric assays was observed. Typical results for the Amplex Red and ABTS activity assays on GOx mutant strains are shown in **Figure 3C and 3D** respectively. Each 96 well plate contained 96 mutant GOx samples that were loaded in identical wells between plates. Upon comparing the two assays, variation in the measured activity of identical samples was observed, as can be seen between **Figure 3C and 3D**, with particularly notable variation for samples located in the lower left side of the plates. The Amplex Red assay routinely identified a greater number of mutants as enzymatically active than were identified by the ABTS assay. Variation in the relative degree of activity obtained for mutants was also observed between assays. Although slight variation was observed in replicates performed using a specific assay, significant variation between the two assays was common. Colorimetric assays generally rely on secondary enzymes, chemical reagents, and/or dyes to report activity. Environmental and chemical sensitivity of these reagents, in addition to batch-to-batch reagent variation, may have contributed to inconsistent results. Additionally, the source of enzyme for these assays was filtered, concentrated, and buffer exchanged spent culture medium which may have contained trace levels of interferants that impacted the colorimetric assays.

### Electrochemical Activity Assay

Promising mutants identified by colorimetric assays were further characterized by an electrochemical assay which yielded precise enzymatic kinetic rate information. Hydrogen peroxide can be directly oxidized at a positively charged platinum electrode yielding one molar equivalent of dioxygen, and two molar equivalents of electrons and protons:

H_2_O_2_ → O_2_+2e^-^ +2H^+^ (3)

The measured current is proportional to the concentration of hydrogen peroxide in solution. The electrochemical measurement of hydrogen peroxide produced by GOx provides a very useful and sensitive method for studying the kinetic properties of GOx. The electrochemical assay chosen is well characterized [17], [18], [19], [20], mediator-less, reproducible, does not depend on secondary enzymes or reporters, and accurate kinetic rate data is obtainable as the measurement is direct and real-time (10 data points collected each second). Previously reported optimized conditions for H_2_O_2_ oxidation on Pt [17], [18], [19], [20] were used for this assay (100 mM sodium phosphate buffer, pH 6.8, and +700 mV vs. Ag/AgCl electrode polarization).

A hydrogen peroxide calibration curve was obtained by measuring the current response for eleven different concentrations of peroxide. Runs were randomized and 3 replicates were performed. A highly reproducible linear current response was observed for concentrations of 1 µM through 1 mM peroxide (**Figure 4A**; R^2^ = 0.9980). The assay sensitivity of 84.3 nA/µmolar H_2_O_2_ was similar to the 84.5 nA/µmolar H_2_O_2_ value reported in an earlier work [21].

Experimental conditions for the GOx activity assay were identical to those described above for the peroxide current response with the exception that glucose at various concentrations was included in the electrolyte solution, instead of hydrogen peroxide. Following application of the positive bias to the rotating Pt electrode and allowing the current to stabilize, 25 µL of a solution containing GOx was injected into the electrochemical cell. Upon sample injection, the solution was instantly mixed with the buffer solution via the rapidly rotating working electrode and an immediate increase in current was measured. The initial rate velocity, *v* ([H_2_O_2_]/min), was obtained from the slope of this current response over the first second (or less) from injection. A detection limit of 1.7 mUnit GOx/mL was achieved, and was adequate for assaying GOx expressed in the yeast system of this work.

Validation of the electrochemical initial rate assay was obtained by performing initial rate velocity measurements at several different glucose concentrations using commercially available GOx from *A. niger* (Biozyme Labs, South Wales, UK). Initial rate velocities were used to calculate the enzymatic activity kinetic parameters. Measured turnover (*k*_cat_) of the commercial GOx was 2.79×10^4^ (µmol product/µmol active subunit/min) compared with 2.75×10^4^ reported by the manufacturer. The Michaels-Menten constant, *K*_M_, was measured to be 14.6 mM. This value was not reported by Biozyme, but is within the range of 3.5–38 mM published for native GOx from *A. niger* under similar conditions [22]. Activity assay positive controls using Biozyme GOx were performed daily prior to assaying the parental or mutant GOx strains.

Enzymatic activity assays of the parental GOx strain and mutant GOx strains were performed using filtered, concentrated, and buffer exchanged spent *S. cerevisiae* culture medium. To ensure that potential interferants that may remain following sample concentration and purification do not contribute to the electrochemical assay response, a sample from a yeast culture in which GOx expression was induced was compared to an uninduced culture sample. While the induced culture sample (+glucose, +protein) showed an immediate increase in current upon injection (**Figure 4B** **,** red trace), the uninduced culture sample (+glucose, –protein) showed no increase in current response throughout the course of the experiment (**Figure 4B** **,** blue grey trace). No increase in current was recorded upon injection of samples prepared from induced cultures in the absence of glucose (– glucose, +protein, **Figure 4B** **,** green trace) showing that galactose metabolism, as opposed to glucose metabolism, did not contribute a response during assay. Therefore, the current response measured from filtered, concentrated, and buffer exchanged culture media samples can be attributed solely to H_2_O_2_ produced from GOx turnover with no contribution from remaining components of the spent media.

### Catalytic Activity of GOx Mutants

As detailed in the Introduction, 15 dissimilar amino acids in the FAD- and substrate-binding sites of *A.* niger were selected for either site-directed mutagenesis to mirror the comparable residues from *Penicillium*, or for saturation mutagenesis. An initial library of nearly 1200 mutants with single point mutations was created. Although the electrochemical assay proved more reliable for collecting kinetic rate data for mutants, the assay time was lengthy and could not be used for high-throughput screening. With the large number of mutants generated, it became necessary to use the more high-throughput colormetric assay as a pre-screen of mutant activity followed by electrochemical analysis of promising mutants. For this pre-screen the ABTS assay was utilized.

Of the 200 mutants colorimetrically screened from the first round of saturation mutagenesis, 14% reported activity comparable to or greater than the parent enzyme, 25% reported activity less than the parental enzyme, and 61% showed no measurable activity. These results are similar to that reported by other directed evolution enzyme studies [9], [10]. Mutants with 2.5 fold higher activity over the parental GOx were selected for sequencing and subsequent kinetic analysis using the electrochemical assay. Due to project budget and time constraints, the remaining mutants were not characterized beyond the initial ABTS activity assay. However, we expect that by comparing kinetic and sequencing data from these less-successful mutants with that obtained from mutants with improved performance, new insights may be obtained that would be useful for future modifications to the GOx active site.

Of the mutants assayed following the first round of saturation mutagenesis, the mutant with the greatest increase in specific activity, as determined by electrochemical assay, had a specific activity of 328±20.6 s^−1^. This represents a 3-fold improvement in specific activity over the parental *A. niger* GOx activity of 112±10.5 s^−1^ (per monomer). The *K*_M_ and specificity constant for this mutant and the parental strain are reported in Table 1. Sequencing of this mutant revealed that it contained a single point mutation from threonine to serine at amino acid 132 (mutant T132S). Both recombinant parental GOx and mutant T132S GOx genes were subjected to two further rounds of site-directed saturation mutagenesis at other targeted residues generating approximately 3500 additional mutants. Of these mutants, 1200 were screened with the ABTS colorimetric assay.

Ultimately, three additional unique mutants with significantly improved catalytic activity over the parent enzyme were identified. The kinetic parameters for these mutants are also reported in Table 1. The mutant strain with the most improved *k*_cat_ (V42Y) showed a 4.5-fold improvement over the parental *A. niger* GOx and a slight improvement over GOx from *P.amag*. However, this mutant had the lowest substrate affinity, 21% lower than the parental stain. Interestingly, a double mutant containing both T132S and V42Y mutations was identified and showed a significant improvement in *K*_M_ over the parental strain with a similar *k*_cat_ to mutant T132S. An additional double mutant was identified (T132S/T56V) which showed the most improved *K*_M_, 38% better than the parental enzyme, but the lowest improvement in *k*_cat_.

### Thermal Stability of GOx Mutants

As the goal of this study was to improve the specific activity of a relatively more stable form of GOx, it was important to ascertain how mutations affected protein stability. A thermal stability assay monitoring enzyme activity over time at 50 °C was validated using GOx from Biozyme. A *T*_70%_ at 50 °C of 10.4 hours was measured. This is similar to *T*_70%_  = 11 hours reported by Kalisz et al. under similar conditions [13], but markedly more stable than *P. amag.* under the same conditions [14].The thermal stability of the four improved mutant GOx strains was also measured and is compared to the parental strain in Table 2. Interestingly, all identified mutants showed a modest improvement in thermal stability over the parental strain. Mutant V42Y, which had the most improved *k*_cat_, also showed the greatest improvement in thermal stability.

## Discussion

Site-directed mutagenesis and directed evolution were used to improve the catalytic activity of recombinant *A. niger* GOx. Mutants were initially screened via a high throughput colorimetric activity assay and further characterized by an electrochemical initial rate assay allowing quantification of kinetic rate parameters. Four mutants with improved catalytic properties were identified out of a library of approximately 4800 mutants, of which only 1400 have so far been screened. These mutants (T132S, T132S/T56V, T132S/V42Y, and V42Y) showed a 3 to 4 fold improvement in specificity constant over the parental strain. The variant with the highest *k*_cat_ (V24Y) showed a 4.5-fold improvement over the parental *A. niger* GOx and slight improvement over GOx from *P.amag*. at the expense of a 21% lower substrate affinity. Also in common with all mutants, V42Y showed a modest improvement in thermal stability (T_1/2_ = 40.3 hours) compared to the parental strain (T_1/2_ = 28.8 hours) and the commercially available enzyme (T_1/2_ = 20.1 hours).

A recent report [23] offers some insight into why the first mutant identified (T132S) may have yielded increased performance. The structure for the flavin-oxygen adduct intermediate was solved for choline oxidase, a closely related GMC oxidoreductase, using a combination of visible spectroscopy and X-ray spectroscopy under cryogenic conditions. In the published structure, the isoalloxazine ring undergoes a large conformational change upon addition of the oxygen adduct. By comparing the crystal structure of GOx (pdb id 1cf3) with the choline oxidase structure (pdb id 2jbv), it was observed that the threonine 132 residue in GOx (an isoleucine in the choline oxidase structure) is directly adjacent to this warped region of the isoalloxazine ring, as shown in **Figure 5**. Replacement of threonine by the less bulky serine could reduce the steric hindrance of this rearrangement while still providing a potential hydrogen bonding donor to the peroxy adduct. As the rate limiting step for GOx turnover under physiological conditions is O_2_ reduction [24], this proposed mechanism of improved oxygen reduction is deemed a reasonable explanation for improved GOx activity resulting from this mutation. However, T132 is also thought to be a ligand for glucose binding [25], and could also have an impact upon glucose oxidation. Experiments measuring dependence of the rate of turnover on O_2_ concentration in this mutant may clarify the mechanism responsible for its enhanced activity, and are planned for future research.

It is less obvious why the modifications at V42Y and T56V have such large impacts upon enzymatic kinetics. As depicted in **Figure** **6**, these residues are in the first region of the FAD-binding domain (residues 20–58), which is the site of ADP-binding and the most conserved region of the FAD-binding site [15]. However, they are not directly involved in binding FAD, or glucose. Their modification must result in more subtle structural rearrangements which may be difficult to quantify without crystal structures or computational modeling of these mutants, which is beyond the scope of this work.

By combining increased catalytic efficiency with modest improvements in stability, the mutant GOx enzymes produced in this work may prove useful as alternatives to the wild-type enzyme in applications ranging from biosensing, the food and wine industry, to the development of high-power biofuel cells.
